# Parent Explanatory Model Personalization as a Method of Reducing Risk for Poor Engagement and Outcomes in PCIT among Culturally Diverse Families

**DOI:** 10.3390/jcm13123541

**Published:** 2024-06-17

**Authors:** Argero Zerr, Kristen McCabe, Dongbowei Zhang, May Yeh

**Affiliations:** 1 Department of Psychology, California State University Channel Islands, 1 University Dr., Camarillo, CA 93012, USA; 2 Department of Psychological Sciences, University of San Diego, 5998 Alcala Park, San Diego, CA 92110, USA; 3 Child and Adolescent Services Research Center, San Diego, CA 92123, USA; 4 Department of Psychology, San Diego State University, 5500 Campanile Dr., San Diego, CA 92182, USA; 5 Department of Psychiatry, University of California, San Diego, 9500 Gilman Dr., La Jolla, CA 92093, USA

**Keywords:** treatment acceptability, personalization, culture, behavioral parent training, parent–child interaction therapy

## Abstract

**Background/Objectives:** Evidence supports the efficacy of Behavioral Parent Training (BPT) interventions such as Parent–Child Interaction Therapy (PCIT) for treating child behavior problems; however, treatment engagement and outcomes vary across ethnic groups. Risk for poor treatment engagement and outcomes may be attributed in part to misalignment between parent explanatory model components (PEMs) and the traditional BPT model, including treatment expectations, etiological explanations, parenting styles, and family support for treatment. The present study aims to examine whether personalized treatment adaptations addressing these PEM–BPT misalignments reduce risk for poor treatment engagement and outcomes. **Methods:** The authors previously utilized the PersIn framework to develop a personalized version of PCIT (MY PCIT) that assesses these PEMs in order to identify families at risk for poor treatment engagement and outcomes. Families were identified as high risk (due to PEM–BPT misalignment) and low risk (meaning those without identified PEM–BPT misalignment) for specific PEMs. Families at elevated risk then received tailored treatment materials designed to improve alignment between the parental explanatory model and the PCIT treatment explanatory model. A recent pilot trial of MY PCIT demonstrated positive treatment outcomes; however, the extent to which adaptations were successful in reducing the underlying risk factors has not yet been examined. **Results:** Findings demonstrate that the personalization approach was effective in reducing indicators of risk, and that families who were initially at high and low risk during pre-treatment reported similar levels of treatment engagement and outcomes by post-treatment. **Conclusions:** The findings suggest that this personalized approach has the potential to reduce risk associated with poor treatment engagement and outcomes for culturally diverse families.

## 1. Introduction

Ethnic minorities are historically underrepresented in psychological research, including clinical trials that examine the efficacy of psychological treatment programs (e.g., [1]). Similarly, ethnic minorities historically have lower rates of service utilization and poorer outcomes when they utilize mental health services (e.g., [2]). Previous research has identified a number of barriers associated with poorer treatment engagement and outcomes, including socio-economic factors (e.g., poverty status, health insurance), logistical factors (e.g., language barriers, transportation difficulties), broader historical factors (e.g., institutional discrimination), as well as individual or familial factors (e.g., beliefs about mental health). The underrepresentation, barriers, and suboptimal outcomes associated with ethnic minority populations in psychological treatments are not restricted to adults but also extend to treatment of youth (e.g., [3]). Treatments designed for addressing behavior problems in young children, such as Behavioral Parent Training programs (BPTs), have demonstrated efficacy [4]; however, research suggests that ethnic minority families have poorer rates of enrollment [5], treatment completion [6,7], and outcomes in BPTs (e.g., [8]). Therefore, culturally adapting treatments has the potential to enhance engagement and outcomes for ethnic minority families in such programs (e.g., [9]).

One approach to improving engagement and outcomes for ethnic minority families in BPTs and other treatments focuses on developing culturally adapted treatments for specific cultural groups. For example, McCabe et al. [10] developed and evaluated a culturally adapted version of PCIT for Mexican American families called Guiando a Niños Activos (GANA). In a pilot randomized controlled trial comparing GANA to standard PCIT and treatment as usual, GANA demonstrated a significant advantage over treatment as usual in terms of parent-reported measures of child symptoms [11]. Other BPT studies have also found support for culturally adapted interventions (e.g., [12,13]). One meta-analysis with 76 different treatment studies, including but not limited to BPTs, found culturally adapted treatments showed a moderate benefit [14]. Another meta-analysis with 21 studies compared culturally adapted and unadapted treatments and found that culturally adapted treatments were significantly more effective [15]. However, not all studies have found culturally adapted treatments to be superior (e.g., [16]). Some research suggests that the effectiveness of cultural adaptations may depend partly on their level of cultural sensitivity. For example, a meta-analysis evaluating 18 group parent training studies found that programs adapted with “deep structure” cultural sensitivity had greater effectiveness than programs adapted with only “basic” or “surface structure” cultural sensitivity [17].

Although there are benefits of tailoring treatments for specific cultural groups, there are several limitations as well [18,19]. Firstly, when tailoring treatments for specific groups, it is difficult to address within-group variability. Families within a specific cultural group can differ according to a number of factors, including immigration history, acculturation, education, experience with marginalization and discrimination, rural/urban living status, traditional cultural values, family support, etc. A second limitation of this approach is that therapists working in the community often have a diverse range of clients from different cultural groups, so developing and training therapists in approaches for each specific group and for multiethnic families may not be feasible.

### 1.1. PersIn Approach

The PersIn approach to treatment adaptation aims to address some of the earlier challenges with cultural adaptation by utilizing assessment-driven personalization across multiple cultural groups [18,19]. In this approach, families complete a pre-treatment assessment, which then triggers the implementation of specific standardized treatment adaptations designed to improve the treatment fit for each family. Compared to earlier treatment research or cultural adaptation approaches, this assessment-driven personalization approach offers a number of benefits. First, the core elements of the treatment are maintained, so the potential threats to treatment fidelity are minimized. Second, the use of a structured assessment and a series of standardized treatment adaptations help to reduce the therapist burden. Utilizing pre-established adaptations as well as personalized, assessment-guided recommendations can reduce therapists’ need to adapt treatments in real time, which may help maintain fidelity to the original treatment. Third, the approach helps therapists address inter-group and intra-group variability, without relying solely upon cultural identity in the selection of treatment adaptations. Fourth, the approach helps to improve the efficiency of the cultural adaptation process by targeting only the components that are warranted.

When applied to BPTs, the PersIn approach proposes that adaptations should involve parent explanatory model components (PEMs) that (a) vary across ethnic groups, (b) impact treatment engagement, change mechanisms, and/or response, and (c) are modifiable [18]. Using these three criteria, a review of the BPT literature identified four specific targets for personalization: (1) treatment expectations, (2) etiological explanations, (3) parenting styles, and (4) family support for treatment [18]. Research evidence supports the notion that misalignment between parental explanatory models and the treatment explanatory model in these areas increases risk for poor treatment engagement and clinical outcomes. In some cases, we identified PEMs that could be targeted for modification, and, in other instances, we identified treatment explanatory model components that could be adapted to better align with the parent explanatory model. Below, we describe each of the four PEMs, how they relate to treatment engagement and outcomes for ethnic minority families, and examples for ways in which treatment modifications may improve the PEM–BPT alignment. 

### 1.2. Parent Explanatory Model Components (PEMs)

First, previous studies have found significant variations in treatment expectations across cultural groups, where African American, Hispanic American, and Asian American participants are less likely than Non-Hispanic White participants to report positive expectations regarding the benefits and outcomes of treatment [20,21]. Research has also demonstrated that treatment expectations are associated with subsequent treatment engagement and outcomes [21,22], including parent pre-treatment expectancies in treatment for youth (e.g., [23]). Positive treatment expectancies have also been linked to stronger therapeutic alliances [24] as well as better treatment outcomes [25]. Thus, if low treatment expectancies are identified and addressed, treatment engagement and outcomes may be improved.

Secondly, existing research has also identified cultural differences in beliefs about the causes of mental health problems (e.g., [26]). For example, when comparing causes of mental health problems between ethnic minority and White participants, ethnic minority participants are more likely to endorse supernatural causes of mental health problems, while White participants are more likely to endorse genetic or biological causes of mental health problems (e.g., [27]). Similarly, child clinical research has also found racial/ethnic differences in parental etiological explanations for child problems [28,29]. This is particularly important given the research showing that parent attributions about the child’s problem predicts help-seeking attitudes, treatment attendance and completion, and treatment outcomes (e.g., [30,31]). Not only have etiological explanations been linked to service utilization, but they have been identified as a potential contributor to ethnic disparities in mental health service utilization (e.g., [32]), and parental etiological explanations partially mediate the relation between race/ethnicity and service utilization [33]. As such, when warranted, increasing the alignment between parental etiological explanations and what the treatment can provide may improve parental engagement in treatment and clinical outcomes.

Thirdly, research has found cross-cultural differences in parenting styles (e.g., [34]). There is also research indicating that parenting styles are linked to treatment completion, including for families involved in BPT. For example, McWey and colleagues [35] found that BPT treatment completers scored higher on authoritative parenting practices, while BPT treatment non-completers scored higher on harsh and inconsistent discipline. Another BPT study found that treatment dropout was predicted by more negative parental talk and less parental praise [36]. Therefore, when implementing BPT techniques, it is plausible to believe that taking parenting attitudes into account, especially those that may seem incompatible with BPT, has the potential to improve treatment acceptability and reduce ethnic disparities in youth mental health care. 

Lastly, previous research has found cross-cultural variation in familial support for treatment. For example, Black parents are twice as likely as White parents to expect disapproval from family members when seeking mental health care for a child [37]. Glazier and colleagues [38] found that ethnic minority participants were significantly more likely than Non-Hispanic White participants to report being afraid that seeking help for mental health problems could result in criticism from family members. Lack of family support for treatment may contribute to mental health stigma, such as feelings of guilt, shame, or embarrassment for seeking help outside the family, including concerns that help-seeking may negatively impact the family’s reputation [39,40,41]. In contrast, perceived familial support is positively associated with treatment acceptability [42]. Fortunately, there is also some indication that family support for treatment is modifiable, particularly by addressing specific concerns and engaging family members throughout the course of treatment [43]. For example, in PCIT, the frequency of therapists’ engagement strategies (such as reaching out, flexible scheduling, providing information, stressing the father’s important role) predicts more treatment involvement by fathers [44]. Thus, incorporating additional family engagement efforts, when warranted, may increase family member support for treatment. 

### 1.3. Present Study

Implementing a treatment personalization approach that attends to those PEMs that have demonstrated relationships to poorer engagement and/or outcomes in BPT for ethnic minority families has the potential to reduce ethnic disparities in youth mental health care. By focusing on individualized needs and tailoring the treatment accordingly, we may be able to provide a more effective and culturally responsive approach for diverse populations. Our recent publication [19] examined the application of this PersIn approach to a specific BPT program—Parent–Child Interaction Therapy (PCIT). This personalized version of PCIT, called MY PCIT, utilized a cultural assessment based on these four PEMs that was used to provide tailored treatment materials designed to reduce risk for poor engagement and outcomes by improving the alignment between parent and treatment explanatory models. Findings from the study demonstrated that the treatment was effective in reducing child behavior problems and improving parental outcomes like parent stress and positive parenting. Although the study found positive outcomes associated with the personalization approach, it is not yet known if or how the approach changed the underlying indicators of risk associated with poor engagement and outcomes. Therefore, the primary aims of the present study are to examine whether (1) treatment adaptations improve risk factors associated with poor engagement and outcomes, and (2) PEM-treatment alignment relates to outcomes. Specifically, we hypothesize that treatment expectations, treatment compatibility with etiological explanations, acceptability of BPT, and/or family support for treatment will improve across the course of treatment for those who were identified to receive adaptations. Secondly, since treatment plans were personalized according to each family’s PEMs, we expect that outcomes will be similar across families who endorsed a particular PEM response, consistent with risk for poor engagement and outcomes at pre-treatment, and those who did not. Exploratory analyses will also examine (1) how different PEMs relate to one another, and (2) if parenting styles changed across the course of treatment. 

## 2. Materials and Methods

### 2.1. Participants

Eligible families were seeking help for a child between the ages of 2 and 7 years with clinically significant behavior problems. Families were excluded if they did not speak English or Spanish, or if the parent or child had a diagnosis of autism or intellectual disability. Participants were 32 caregivers (referred to hereafter as parents; *M* = 37.94 years, 94% female) of children aged 2–7 years (*M* = 4.66 years, 34% girls) with clinically significant behavior problems. Child ethnicity included 28% Hispanic or Latinx, 22% non-Hispanic White, 16% Asian American, 13% Black or African American, and 22% multi-ethnic youth. Multi-ethnic youth were Non-Hispanic White (100%), Asian American/Pacific Islander American (43%), Black/African American (43%), and Latinx (29%). Parent education included 6% less than high school, 3% completed high school, 47% some college, 19% college graduate, and 25% graduate degree. Monthly household income was less than $5000 (38%), $5000 to $9999 (25%), $10,000 or greater (22%), or not reported (16%). Marital status for the primary parent was 66% married, 28% single, and 6% divorced. In regard to parent generation in the United States, 25% were 1st generation, 34% were 2nd generation, 3% were 3rd generation, 9% were 4th generation, and 28% were 5th generation or higher. Parents completed their assessments in English (*n* = 31) or Spanish (*n* = 1). 

### 2.2. Procedures

Families were recruited through child mental health providers, public advertisements, and word of mouth. Once screened, eligible families were invited to the university lab to review consent procedures and complete the pre-treatment assessment. This assessment included a structured observational parent–child interaction task and a series of parent report surveys, including the personalization assessment tool. Therapists were then sent a personalized treatment plan that indicated the specific adaptations that were triggered by the parent’s assessment, including the personalized treatment materials. Families then started the treatment program in community settings (e.g., children’s hospital outpatient clinic) or at the university lab. Therapists were master’s- and doctoral-level therapists trained in PCIT and supervised weekly by the second author, who was a PCIT Within Agency Trainer. During the course of treatment, parents completed two brief phone assessments: one after the Child-Directed Interaction (CDI) Teach session and the other after the Parent-Directed Interaction (PDI) Teach session. Families returned to the university lab to complete a post-treatment assessment, which consisted of a structured parent–child interaction task and parent report surveys. Parents were compensated $100 for each of the two in-person assessments and $10 for each of the two phone assessments. A CONSORT flow diagram of participant involvement has been previously reported [19]. 

### 2.3. Standard Treatment

PCIT is an evidence-based BPT intervention for child behavior problems [45,46]. Treatment includes approximately 14 weekly (1–2 h) sessions, divided into two phases. During the first phase, CDI, parents learn and practice ways to develop a warm and positive relationship with their child by using selective attention to reinforce positive behaviors rather than negative attention-seeking behaviors. These skills are practiced for several sessions until the parent reaches graduation criteria to move to the second phase. The second phase, PDI, focuses on reducing problem behaviors by improving consistent discipline. Once parents have achieved graduation criteria for both CDI and PDI skills, the family graduates from PCIT. 

### 2.4. Treatment Personalization Process

In addition to delivering the core elements of PCIT, a computerized assessment program for PEMs was utilized to identify parents at high risk for poor engagement or outcomes. The program scored each measure (described below) and produced a corresponding list of treatment adaptations drawn from a menu of 39 different materials, including handouts, videos, therapist discussion guides, and phone calls (see [18] for more detail). The overall goal of the treatment personalization process was to reduce risk factors associated with poor treatment engagement and response by improving the alignment between the treatment explanatory model (TEM) and the parent explanatory model. Some adaptations addressed this by focusing on influencing the PEMs, while other adaptations focused on the presentation of the TEM.

### 2.5. Parent Explanatory Model Components (PEMs)

#### 2.5.1. PEM 1: Treatment Expectations

The Outcome Expectancies Scale includes 9 items that were selected and modified from the Parent Expectancies for Therapy Scale [21] to assess parent expectations regarding treatment effectiveness. Items are measured on a 5-point response scale ranging from 1 “do not believe/not at all” to 5 “strongly believe/a great deal”. Items are summed to compute the total expectancies scale. Treatment adaptations were triggered for families who endorsed at least one low outcome expectancy (by selecting 1 “do not believe/not at all” or 2 “doubt/very little”). Cronbach’s alpha for the present study was good (α = 0.82).

The Accuracy/Role Expectations Scale includes 8 items about the structure and format of treatment. Items were selected and modified from the Parent Expectancies for Therapy Scale [21]. Each item has a true or false response scale, and the total accuracy of expectations is computed by taking the sum of accurate responses. Treatment adaptations were triggered for families who endorsed at least one inaccurate expectation. Cronbach’s alpha for the present study was acceptable (α = 0.71).

Third, the Parent Motivation Inventory (PMI) included 25 items assessing parent motivation for treatment [47]. Items utilize a 5-point scale ranging from 1 (strongly disagree) to 5 (strongly agree) and are summed to compute total parent motivation. The PMI has previous evidence of reliability and validity [47]. Families with low motivation (as indicated by an average item-level score of less than 4 “agree”) were assigned treatment adaptations. Cronbach’s alpha for the present study was excellent (α = 0.93). 

#### 2.5.2. PEM 2: Etiological Explanations

First, a shortened and modified version of the Beliefs about the Causes of Child Problems Scale [48] assessed parent beliefs about the cause of their child’s problems. Nine items utilized a yes/no response scale for parents to report on a range of possible etiological explanations as causes, at least in part, of their child’s problems. At the end of the measure, parents have the opportunity to mention any other beliefs not yet mentioned, and then a last item asks parents to indicate which of the endorsed items they believe is the primary cause of their child’s problems. The original measure has previous psychometric support [29,49]. In order to assess treatment compatibility with etiological explanations, the parent is also asked two questions regarding this primary cause of their child’s problems: to what extent do they believe that this treatment program can help their child’s behavior problems with this primary cause, and to what extent do they believe that this treatment program can help other children with behavior problems with this primary cause. These items utilize a scale of 0 “strongly disagree” to 10 “strongly agree”, and items are summed to reflect the treatment compatibility with etiological explanations (α = 0.95). 

At pre-treatment, parents reported believing that the main cause of their child’s behavior problems was due to the following factors: personality or temperament (*n* = 12), ADHD (*n* = 6), biological, genetic, or medical issues (*n* = 5), trauma (*n* = 4), parenting (*n* = 3), or other causes (*n* = 2). Treatment adaptations were triggered for families who indicated that the primary cause of their child’s behavior problem was something other than parenting. For this PEM, the goal was not to change the parent’s etiological explanation but to increase the PEM–TEM alignment through adaptations that relayed the relevance of the treatment to the parent’s beliefs.

#### 2.5.3. PEM 3: Parenting Styles

The Acceptability of Behavioral Parent Training Techniques Questionnaire (modified from [10]) included 5 items assessing acceptability of behavioral parenting training techniques: praise, play, timeout, ignoring minor misbehavior, and reducing criticism. Items utilize a 5-point scale ranging from 1 (not at all comfortable) to 5 (very comfortable). Items were summed together to reflect total acceptability of BPT techniques; however, internal reliability was low (Cronbach’s α = 0.04), suggesting that acceptability of BPT may be heterogeneous. As such, data analyses examining change in acceptability of BPT techniques utilize the sum score, as well as the individual items. 

The Parenting Styles and Dimensions Questionnaire (PSDQ) [50] was utilized to assess parenting styles informed by Baumrind’s model. Based on prior psychometric evidence, the current study utilized the short-form version of the Authoritarian Scale [51] and the long-form version of the Permissive Scale. Each item asks parents to rate how likely “good parents” are to use each parenting behavior on a response scale from 1 (strongly disagree) to 5 (strongly agree). Cronbach’s alpha for the present study was acceptable for the authoritarian (α = 0.81) and permissive subscales (α = 0.71).

Treatment adaptations were triggered for families who scored high on authoritarian or permissive parenting attitudes, as indicated by mean item-level scores of greater than two. If a participant scored above two on both the Authoritarian and Permissive scales, then treatment adaptations addressed the parenting style with the higher mean score. For this component, the goal was not to change the parent’s parenting style, but to improve the PEM–TEM alignment through adaptations that emphasized the importance of treatment in the context of their particular parenting style. Additional treatment adaptations were triggered for families who also indicated low acceptability of any BPT technique (as indicated by any item-level scores of 1 “not all comfortable” to “3 “neutral”). Again here, the goal was not to change the parent’s parenting style, but to improve treatment acceptability in the context of their parenting style. Participants also completed a slightly modified version of the interdependence subscale of the Self-Construal Scale [52]. However, because all families scored higher than our predetermined threshold for adaptations and thus received associated adaptations, no comparisons could be made related to receipt of the relevant adaptations; thus, findings from this measure are not included in the present study.

#### 2.5.4. PEM 4: Family Support for Treatment

Family support was measured using a scale (modified from [10]) that involved an initial two items assessing which family members were involved in the child’s care or influencing parenting decisions, and then assessing the perceived support of those family member(s) for the child’s involvement in treatment. Support for the child’s involvement in treatment was measured on a 5-point scale ranging from 1 (very negative) to 5 (very positive). For each family member who was involved in care/influenced parenting decision-making for the child and not supportive of the child’s treatment, parents were asked two additional questions about the family member’s specific concerns and ways to address the concern. Treatment adaptations were warranted for parents with low family support, as indicated by having at least one involved family member with neutral or negative attitudes about the program.

### 2.6. Post-Treatment Outcomes

Engagement. The Parent Participatory Engagement Measure (PPEM) is a 5-item parent report measure assessing parent participatory engagement in the most recent therapy appointment [53]. Items were modified from the original measure to fit the present study’s focus upon PCIT and to refer to participation in the program as a whole. Two additional items were added to assess PCIT skills practice outside of session. Each of the 7 items is rated on a 5-point scale ranging from (1) not at all to (5) very much. Items are summed to represent total treatment engagement. The alpha for the adapted 7-item scale used in the present study was good (α = 0.88 at post-treatment). 

Treatment Satisfaction. The Therapy Attitude Inventory (TAI) is a 10-item measure [54] that assesses parent satisfaction with treatment. Items utilize a 5-point response scale from 1 to 5 and are summed to comprise total satisfaction level. The measure has previous psychometric support [55,56], and the alpha for the present study was good (α = 0.89 at post-treatment).

Child Behavior Problems. The Eyberg Child Behavior Index (ECBI) [57] is a 36-item parent report survey assessing behavior problems in young children. Items utilize a 7-point response scale ranging from (1) never to 7 (always) and are summed to comprise total behavior problems, with sums over 130 representing clinically significant levels. The ECBI is a frequently used measure, with previous evidence of reliability and validity in English and Spanish [57,58]. The alphas for the present study were good: α = 0.88 at pre-treatment, α = 0.97 at post-treatment.

Treatment Completion. Treatment completion was indicated by graduation from PCIT. In order to graduate from PCIT, parents must demonstrate reaching graduation criteria for CDI and PDI parenting skills, child behavior problems on the ECBI must score at or below 114, and parents must report that the child’s behavior problems have been resolved.

## 3. Results

### 3.1. Data Analytic Plan

Data analyses focus on examining the change in specific PEMs across time and the relation between PEM adaptations and treatment engagement, satisfaction, clinical outcomes, and treatment completion. Analyses focus on comparing change in specific PEMs across time for parents with and without treatment adaptations corresponding to that PEM. Specifically, paired samples *t*-tests were used to compare means on specific PEMs across time, and Cohen’s d was calculated to estimate the corresponding effect sizes. Treatment adaptations corresponding to treatment expectations and etiological explanations were primarily delivered in the first few sessions of treatment, especially within the first introductory session and at the beginning of the first treatment phase (CDI). As such, evaluation of the change in treatment expectations and treatment compatibility with etiological explanations compares levels between the pre-treatment assessment and the time 2 assessment, which occurred after the first session of CDI (CDI Teach). In contrast, treatment adaptations corresponding to parenting styles and family support were delivered throughout the course of treatment, if indicated, so change in parenting styles and family support compared levels between the pre-treatment and post-treatment assessments.

Additional analyses also compared treatment outcomes between families with and without treatment adaptations. Specifically, independent samples *t*-tests were used to compare post-treatment means on engagement, satisfaction, and child behavior outcomes between families with and without treatment adaptations, while chi-square analyses compared rates of treatment completion between families with and without treatment adaptations. Additional exploratory analyses also utilized Pearson correlations to estimate the relation among the PEMs. Data analyses were completed using IBM SPSS Statistics Version 29 [59]. 

### 3.2. Preliminary Analyses

Table 1 displays the number of participants, means, standard deviations, and correlations for the PEMs at the pre-treatment assessment, for the present study. The table also includes the number of participants who completed each measure at pre-treatment, along with the number of participants who were triggered to receive corresponding adaptations based on these pre-treatment scores. The number of PEMs that were triggered for adaptation (outside of the interdependence subscale) varied across participants: one family triggered adaptations for only 1 PEM, 13 families triggered adaptations for two of the PEMs, 18 families triggered adaptations for three of the PEMs, and none of the families triggered adaptations for all four PEMs. 

Of the 32 families who completed the pre-treatment assessment and were assigned to begin therapy, 29 (91%) completed the time 2 assessment, and 28 (88%) returned for their post-treatment assessment. Missing data analyses compared families with at least one missing assessment (*n* = 9; 28%) to families who attended all 3 assessments (*n* = 23; 72%) on key sociodemographic and analytic variables. There were no significant differences between families with and without missing assessments on pre-treatment measures of child age, parent age, household income, parent generation, outcome expectancies, accuracy of expectations, parent motivation, acceptability of BPT, authoritarian parenting, permissive parenting, or treatment compatibility with etiological explanations. However, family support for treatment did vary across participants who missed at least one assessment. Specifically, the attitude of involved family members was significantly more supportive of treatment among those missing at least one assessment (*M* = 4.86) compared to those who attended all assessments (*M* = 3.80, *p* = 0.001). Also, participants who missed at least one assessment had fewer family members with negative or neutral attitudes (*M* = 0) compared to those who attended all assessments (*M* = 0.35, *p* = 0.008). Subsequent data analyses utilized a pairwise deletion approach, where each analysis included cases with available data corresponding to that particular analysis.

### 3.3. Examining Change in PEMs

#### 3.3.1. PEM 1: Treatment Expectations

Overall, expectations for treatment were fairly high at pre-treatment, but 60% (*n* = 19) of parents still endorsed at least one low treatment expectation. As shown in Table 2, families with initially low outcome expectancies showed significant improvement in expectancies by time 2 following receipt of treatment expectation adaptations (*t*[16] = 2.64, *p* = 0.02, *d* = 0.64). For families who did not meet the threshold for treatment expectation adaptations, overall outcome expectancies did not show significant change by time 2 (*t*[11] = 1.47, *p* = 0.17, *d* = 0.43). Pre-treatment scores on the accuracy of expectations were also high overall; however, half of the sample (*n* = 16) endorsed at least one inaccurate expectation. Between pre-treatment and time 2, there was no significant change in the total accuracy of expectations among parents who initially reported inaccurate expectations and received corresponding adaptations (*t*[14] = 1.61, *p* = 0.13, *d* = 0.42; see Table 2). However, parents who did not meet the threshold for these adaptations showed a significant decrease in the total accuracy of expectations by time 2 (*t*[13] = 2.69, *p* = 0.02, *d* = 0.72). At pre-treatment, overall parent motivation was moderately high for this treatment-seeking sample, but 6 parents did report low motivation. Between pre-treatment and time 2, parents with these initially low levels of parent motivation showed a significant increase in motivation by time 2 (*t*[5] = 2.68, *p* = 0.04, *d* = 1.09), after receipt of corresponding adaptations. In contrast, parents who did not meet the threshold for these adaptations showed a marginal increase in motivation by time 2 (*t*[21] = 1.98, *p* = 0.06, *d* = 0.42).

#### 3.3.2. PEM 2: Etiological Explanations

In regard to treatment compatibility with etiological explanations, parents largely agreed that the program could help their child with this main cause (*M* = 8.09 of 10), and agreed that the program could help other children with this main cause (*M* = 8.13 of 10). At pre-treatment, the majority of parents (*n* = 29) indicated that they believed the main cause of their child’s problems was a factor other than parenting. By time 2, overall scores on treatment compatibility with etiological explanations remained high for parents who initially reported that the main cause of their child’s behavior problems was due to a factor other than parenting, and who had received corresponding adaptations (*t*[24] = 0.55, *p* = 0.59, *d* = 0.11; see Table 2).

#### 3.3.3. PEM 3: Parenting Styles

At pre-treatment, parents reported that, on average, they were somewhat comfortable with BPT techniques; however, 24 parents reported discomfort with at least one BPT technique. By post-treatment, parents’ attitudes towards BPT techniques showed significant improvement among parents who reported discomfort with at least one BPT technique and had received adaptations (*t*[19] = 5.15, *p* < 0.001, *d* = 1.15), as well as parents who did not meet the threshold for those adaptations (*t*[7] = 2.65, *p* = 0.03, *d* = 0.94; see Table 3).

#### 3.3.4. PEM 4: Family Support for Treatment

At pre-treatment, parents reported, on average, that involved family members had somewhat positive attitudes about their involvement in the program, with approximately one quarter of parents (*n* = 7) indicating they had an involved family member with a negative or neutral attitude. By post-treatment, families with initially low levels of family support for treatment, who had received adaptations, showed significant reduction in the number of unsupportive family members (*t*[6] = 4.58, *p* = 0.004, *d* = 1.73) and improvement in the overall attitude of involved family members (*t*[6] = 4.34, *p* = 0.005, *d* = 1.64). On average, these families with adaptations reported one fewer unsupportive family member by post-treatment, with only one family reporting an unsupportive family member. In contrast, families who initially did not meet the threshold for these adaptations showed a significant increase in the number of unsupportive family members by post-treatment (*t*[20] = 2.65, *p* = 0.02, *d* = 0.58) and a marginal decrease in the average attitude of involved family members (*t*[15] = 2.03, *p* = 0.06, *d* = 0.51).

### 3.4. Treatment Engagement, Satisfaction, and Outcomes

Post-treatment parent participation engagement scores ranged from 8 to 35, with an average of 28.78 (*SD* = 5.75), indicating that parents were, on average, between “quite a bit” and “very much” engaged. Treatment satisfaction ranged from 31 to 50 (*M* = 43.64, *SD* = 4.92), indicating that, on average, parents were between “somewhat” and “very satisfied” at post-treatment. At post-treatment, average child behavior scores on the ECBI intensity scale were within the normative range (see [19]). Post-treatment levels of parent participatory engagement, treatment satisfaction, and child behavior problems were not significantly different between families with and without adaptations (all *p*s > 0.05). Specifically, post-treatment levels of parent participatory engagement, treatment satisfaction, and child behavior problems were not significantly different between families with and without adaptations related to treatment expectations (*p*s > 0.05), inaccurate expectations (*p*s > 0.05), parent motivation (*p*s > 0.05), etiological explanations (*p*s > 0.05), acceptability of BPT (*p*s > 0.05), authoritarian or permissive parenting attitudes (*p*s > 0.05), or family support for treatment (*p*s > 0.05).

In total, 50% (*n* = 16) of families completed the entire treatment and reached PCIT graduation criteria, whereas 50% (*n* = 16) of families did not complete treatment (see [19]). Many families who did not fully complete treatment saw statistically and clinically significant improvement, and families reported not completing treatment due to a variety of factors, including moving out of the area. When examining findings related to specific PEMs, treatment completion rates were not significantly different between those with and without adaptations for treatment expectations (*Χ*^2^[1] = 1.17, *p* = 0.28) or inaccurate expectations (*Χ*^2^[1] = 0.50, *p* = 0.48). However, other cell sizes were too small to compare treatment completion rates between families with (43–57%) and without (33–67%) adaptations for parent motivation, etiological explanations, acceptability of BPT, authoritarian or permissive parenting attitudes, or family support for treatment.

### 3.5. Exploratory Analyses

#### 3.5.1. Relations among PEMs

As displayed in Table 1, most PEMs were not significantly correlated with each other, demonstrating variability in parent perceptions of treatment across different domains. Different aspects of the treatment expectations PEM were not significantly correlated with one another (*r*s = −0.11 to 0.28), nor were they significantly correlated with parenting styles (*r*s = −0.32 to 0.20) or with family support for treatment (*r*s = −0.25 to 0.14). However, parent motivation was positively correlated with treatment compatibility with etiological explanations, suggesting that motivation may be higher when parents believe that the treatment is a good fit for the main cause of their child’s problems. Treatment compatibility with etiological explanations was not significantly correlated with any other PEMs (*r*s = −0.25 to 0.28). Within the parenting styles PEM, permissive and authoritarian parenting styles were positively correlated with one another. Otherwise, different aspects of the parenting styles PEM were not significantly correlated with other PEMs (*r*s = −0.32 to 0.23). Lastly, measures of family support for treatment were not significantly correlated with other PEMs (*r*s = −0.30 to 0.23), although there was an expected negative correlation between the average attitude of involved family members and the number of unsupportive family members.

#### 3.5.2. Parenting Styles

Exploratory analyses also examined change in parenting attitudes from pre- to post-treatment. By post-treatment, both authoritarian (*t*[27] = 7.36, *p* < 0.001, *d* = 1.39) and permissive attitudes (*t*[27] = 2.61, *p* = 0.015, *d* = 0.49) showed significant reductions for the overall sample (see Table 3). Parents who were initially high on authoritarian or permissive parenting attitudes and who had received corresponding adaptations saw significant decreases in both authoritarian (*t*[21] = 7.35, *p* < 0.001, *d* = 1.57) and permissive attitudes (*t*[21] = 2.91, *p* = 0.008, *d* = 0.62) by post-treatment. Meanwhile, for parents who did not meet the threshold for these adaptations, there was a marginal decrease in authoritarian attitudes (*t*[5] = 2.56, *p* = 0.05, *d* = 1.04) and no significant change in permissive attitudes (*t*[5] = 0.75, *p* = 0.49, *d* = 0.31).

## 4. Discussion

The purpose of this study was to examine a personalized treatment approach to attending to parent explanatory model components (PEMs) that have been associated with poorer engagement and outcomes among a sample of culturally diverse families in PCIT. Previous research has demonstrated that ethnic minorities have poorer treatment engagement and outcomes in mental health services [60]. Additional research suggests that variability in PEMs and/or their misalignment with treatment explanatory model components might help to explain ethnic disparities in treatment, particularly treatment expectations, etiological explanations, parenting styles, and family support for treatment (e.g., [21,33,38]). A personalized approach may help to attend to cultural and familial variability in these PEMs in order to improve treatment engagement, satisfaction, and outcomes for ethnic minority families. A prior study [19] found positive treatment outcomes associated with this approach; however, we had not yet examined whether the effects of the personalization process improved the alignment between the TEM and PEM. Generally, findings from the present study support the feasibility and potential of using this assessment-driven personalization approach to improve indicators of risk associated with poor treatment engagement and outcomes.

As hypothesized, the PersIn approach produced significant changes in specific PEMs across the course of treatment for those who received associated adaptations, demonstrating the feasibility of adapting these PEMs with the use of personalized treatment materials. Specifically, treatment adaptations were able to improve treatment expectations, including outcome expectancies and parent motivation. Parents who received treatment adaptations also showed improvement in the acceptability of BPT as well as family support. It is interesting to note that families without adaptations actually showed a decline in family support by post-treatment, suggesting that family support has the potential to decrease throughout treatment, even among parents who did not initially report low family support. It is possible that these families initially believed that their families would be supportive but received resistance or skepticism once they started to employ new parenting techniques at home (e.g., ignoring minor misbehavior, utilizing timeout). This highlights the need for assessing family support throughout the course of treatment and possibly proactively addressing potential concerns with family support.

Utilizing this personalized approach to guiding the delivery of treatment adaptations was also related to positive outcomes. Not only were treatment outcomes similar to other treatment studies [19], but findings in the present study demonstrate that the delivery of treatment adaptations eliminated expected post-treatment outcome differences. Specifically, parent participatory engagement, treatment satisfaction, child behavior problems, and treatment completion were all similar for those with scores consistent with increased risk for poor engagement and outcomes and those with scores consistent with lower risk across the four PEMs. These findings suggest that the personalization approach has the potential to eliminate potential barriers to treatment engagement or outcomes that would otherwise be evident.

Correlations indicated that pre-treatment scores on the PEMs were minimally related to one another, demonstrating heterogeneity in and relative independence across parental attitudes about treatment in our culturally diverse sample. In addition, the pattern of PEMs triggered for adaptation also suggests variability across families. These findings highlight the need for identifying and personalizing specific aspects of treatment based on key PEMs, rather than adapting all treatment aspects universally. There were a few exceptions, where pre-treatment PEMs were significantly correlated with one another. Pre-treatment levels of parent motivation were positively related to treatment compatibility with etiological explanations, which suggests that parents might be more motivated when they believe that the treatment aligns with their etiological explanations. Permissive and authoritarian parenting attitudes were positively correlated with one another, which has also been found in other studies (e.g., [55]). This inter-correlation between authoritarian and permissive parenting indicates a complicated relationship between parenting attitudes. Exploratory analyses also demonstrated changes in parenting styles across the course of treatment, particularly for parents who were initially authoritarian or permissive and given corresponding treatment adaptations. These adaptations were designed to improve the alignment between the treatment approach and the parent’s parenting style, and not necessarily to change the parenting style directly. That being said, these findings suggest that tailoring the program materials to account for parenting style has the potential to facilitate changes in parenting attitudes.

In sum, these findings are critical to our understanding of how personalized treatment approaches work, especially since there have been few studies examining if or how treatment adaptations really address underlying treatment attitudes. Importantly, findings highlight the potential for personalized treatment approaches to address risk factors associated with poor treatment engagement and outcomes. This approach may be particularly important for serving ethnic minority families who may be at higher risk for poor treatment engagement or worse treatment outcomes.

### 4.1. Strengths and Limitations

The present study addresses an important gap in the existing literature, specifically by examining if an approach to personalizing an intervention is related to change in factors associated with poor treatment engagement and outcomes. The PersIn approach takes a systematic and scaffolded approach to identifying and addressing potential barriers. This type of approach has the capability to better address inter- and intra-group variability in families, and has great potential for scalability. For example, this approach could be implemented in heterogeneous populations, including across families with varying degrees of treatment experience and acceptability. This type of adaptability has the potential to better serve clinicians and families in diverse communities.

Although therapists are often trained in more general ways to assess cultural variation and barriers to treatment, the PersIn approach involves a structured series of assessment tools and corresponding treatment adaptations, therefore reducing the burden associated with tailoring treatments. The MY PCIT program included a digital version of this assessment, which was programmed to automatically score each measure, identify which PEMs warrant personalization, and produce a therapist report that includes the corresponding treatment adaptations. Therefore, the digital aspect of this assessment process eliminates the time it takes to score the measures, apply thresholds, and identify corresponding treatment materials.

Two primary limitations with the present study are the small sample size and lack of experimental design. Although the sample size was sufficiently powered for conducting many of the repeated measures comparisons, the study was underpowered for cross-participant comparisons. Sample sizes were too small to examine potential moderators and mediators of change. Moreover, since the study was a pilot feasibility study that lacked experimental design and a control group, we cannot determine if the cross-time changes in PEMs were solely due to the personalization approach. Families may have naturally improved on these factors across the course of time, regardless of personalization. In order to address these factors, a larger experimental design is needed, such as one where families are randomly assigned to receive either a standard treatment or to receive personalized treatment adaptations. Likewise, a larger study would allow for analysis of individual differences, including sociodemographic or psychological factors that may moderate the treatment effect. Lastly, although missingness was not related to any other analytic or sociodemographic variable, it was related to levels of family support. It is unclear why parents who reported more family support at pre-treatment would be less likely to complete one of their subsequent assessments, but findings should be interpreted with this in mind.

### 4.2. Future Directions

Future directions in this area should address several key areas of investigation. Firstly, there is a need for experimental design to directly compare the personalized and non-personalized versions of treatment. This will allow for a more comprehensive understanding of the differential impact and efficacy of personalized treatment programs. Additionally, further research is warranted to identify the specific threshold that should trigger a treatment adaptation within the context of personalized treatments. Understanding the optimal point at which to introduce personalized strategies can enhance treatment outcomes. Moreover, it is essential to explore how to effectively address personalization when multiple caregivers are involved in the treatment process in order to accommodate diverse family structures. For example, there is emerging research aimed at better understanding ways to engage male caregivers in parenting interventions [61], with recognition that parental responsibilities are shifting and these societal changes warrant an update to the ways in which father engagement is addressed (e.g., [44,62]). This may be particularly important given that maintenance of PCIT treatment gains is better for mothers from involved-father families compared to families where fathers were absent [63]. Similarly, interventions with older children and adolescents may need to identify the best way to address perceptions from both caregivers and youth.

It is worth noting that the personalization model relied on assigning treatment adaptations based on the parent’s pre-treatment assessment. An important avenue for future research involves applying the personalization approach to other BPTs and also to other treatment modalities, as, theoretically, the PersIn approach can be applied to personalize any treatment [18,19]. This expansion will allow for a broader examination of the generalizability and effectiveness of personalized interventions across different therapeutic frameworks. This research can help to improve understanding of the factors affected by this personalized intervention process in order to improve treatment engagement and effectiveness for culturally diverse families. Collectively, addressing these research directions will advance our understanding and implementation of personalized treatments in the field of psychology.

## Figures and Tables

**Table 1:** Number of participants, means, standard deviations, and correlations among PEMs at pre-treatment.

		***N***	***N* with Adaptations**	***M***	***SD***	**1**	**2**	**3**	**4**	**5**	**6**	**7**	**8**	**9**
PEM 1: Treatment Expectations		32	19											
1	Outcome expectancies	32	1	38.63	3.71									
2	Accuracy of expectations	32	16	7.22	0.94	−0.11								
3	Parent motivation	31	8	109.52	10.33	0.28	−0.02							
PEM 2: Etiological Explanations		32	29											
4	Treatment compatibility with etiological explanations	32	29	16.22	3.43	−0.19	0.23	0.44 *						
PEM 3: Parenting Styles		32	26											
5	Acceptability of BPT techniques	32	22	20.72	1.99	−0.12	0.14	0.20	0.28					
6	Authoritarian parenting attitudes	32	10	24.63	6.36	−0.32	−0.05	−0.01	0.14	−0.06				
7	Permissive parenting attitudes	32	16	37.81	3.59	−0.07	0.02	−0.27	−0.25	0.12	0.43 *			
PEM 4: Family Support for Treatment		32	7											
8	Number of unsupportive family members	32	7	0.25	0.51	−0.05	0.02	0.14	−0.07	0.23	−0.20	0.01		
9	Attitude of involved family members	26	7	4.08	1.06	−0.03	−0.25	−0.15	0.20	0.09	−0.04	−0.30	−0.71 **	

* *p* < 0.05, ** *p* < 0.01.

**Table 2:** Means and effect sizes for treatment expectations and etiological explanations across pre-treatment and time 2.

	With Adaptations			Without Adaptations		
	Pre	Time 2	*d*	Pre	Time 2	*d*
Treatment Expectations						
Outcome expectancies	38.00	40.94 *	0.64	39.00	37.42	0.43
Accuracy of expectations	6.53	7.00	0.42	8.00	7.29 *	0.72
Parent motivation	101.33	116.00 *	1.09	113.18	116.50 ^†^	0.42
Etiological Explanations						
Treatment compatibility with etiological explanations	16.52	16.92	0.11	17.33	15.67 ^a^	^a^

Paired samples *t*-tests compared the means across pre-treatment and time 2; subscripts correspond to the following *p*-values: ^†^ *p* < 0.1, * *p* < 0.05, ^a^ statistical comparison not conducted due to a small sample (*n* = 3).

**Table 3:** Means and effect sizes for parenting styles and family support across pre- and post-treatment.

	With Adaptations			Without Adaptations		
	Pre	Post	*d*	Pre	Post	*d*
Parenting Styles						
Acceptability of BPT techniques	19.75	22.4 ***	1.15	22.88	23.88 *	0.94
Authoritarian parenting attitudes	26.00	19.82 ***	1.57	19.83	17.00 ^†^	1.04
Permissive parenting attitudes	38.63	35.77 **	0.62	35.33	32.33	0.31
Family Support for Treatment						
Number of unsupportive family members	1.14	0.14 **	1.73	0.00	0.33 *	0.58
Attitude of involved family members	2.67	4.50 **	1.64	4.53	4.16 ^†^	0.51

Paired samples *t*-tests compared the means across pre- and post-treatment; subscripts correspond to the following *p*-values: ^†^ *p* < 0.1, * *p* < 0.05, ** *p* < 0.01, *** *p* < 0.001.

## Data Availability

Some data used in the preparation of this manuscript are available from the National Institute of Mental Health (NIMH) Data Archive (NDA). NIMH NDA is a collaborative informatics system created by the NIMH to provide a national resource to support the sharing of federally funded data for accelerating research. Dataset identifier: 10.15154/pkt2-ry29.
